# Design and Evaluation of Stand-to-Sit and Sit-to-Stand Control Protocols for a HIP–Knee–Ankle–Foot Prosthesis with a Motorized Hip Joint

**DOI:** 10.3390/bioengineering13010048

**Published:** 2025-12-31

**Authors:** Farshad Golshan, Natalie Baddour, Hossein Gholizadeh, David Nielen, Edward D. Lemaire

**Affiliations:** 1 Department of Mechanical Engineering, University of Ottawa, Ottawa, ON K1N 6N5, Canada; nbaddour@uottawa.ca; 2 Faculty of Medicine, University of Ottawa, Ottawa, ON K1H 8M5, Canada; hgholizadeh@uottawa.ca (H.G.); elemaire@ohri.ca (E.D.L.); 3 Ottawa Hospital Research Institute, Ottawa, ON K1H 8L6, Canada; 4 Nielen Orthotics and Prosthetics, Ottawa, ON K2A 2C5, Canada; dnielencpo@gmail.com

**Keywords:** power hip, impedance-control, sitting, standing up, kinetics, kinematics

## Abstract

**Background:** Sitting and standing with conventional hip–knee–ankle–foot (HKAF) prostheses are demanding tasks for hip disarticulation (HD) amputees due to the passive nature of current prosthetic hip joints that cannot assist with moment generation. This study developed a sitting and standing control strategy for a motorized hip joint and evaluated whether providing active assistance reduces the intact side demand of these activities. **Methods:** A dedicated control strategy was developed and implemented for a motorized hip prosthesis (Power Hip) compatible with existing prosthetic knees, feet, and sockets. One HD participant was trained to perform sitting and standing tasks using the Power Hip. Its performance was compared with the participant’s prescribed passive HKAF prosthesis through measurements of ground reaction forces (GRFs), joint moments, and activity durations. GRFs were collected using force plates, kinematics were captured via Theia3D markerless motion capture, and joint moments were computed in Visual3D. **Results:** The Power Hip enabled more symmetric limb loading and faster stand-to-sit transitions (1.22 ± 0.08 s vs. 2.62 ± 0.41 s), while slightly prolonging sit-to-stand (1.69 ± 0.49 s vs. 1.22 ± 0.40 s) compared to the passive HKAF. The participant exhibited reduced intact-side loading impulses during stand-to-sit (4.97 ± 0.78 N∙s/kg vs. 15.06 ± 2.90 N∙s/kg) and decreased reliance on upper-limb support. Hip moment asymmetries between the intact and prosthetic sides were also reduced during both sit-to-stand (−0.18 ± 0.09 N/kg vs. −0.69 ± 0.67 N/kg) and stand-to-sit transitions (0.77 ± 0.20 N/kg vs. 2.03 ± 0.58 N/kg). **Conclusions:** The prototype and control strategy demonstrated promising improvements in sitting and standing performance compared to conventional passive prostheses, reducing the physical demand on the intact limb and upper body.

## 1. Introduction

Amputees who use lower extremity prostheses, particularly those with hip disarticulation (HD) or hemipelvectomy (HP) amputations, face considerable challenges in common daily activities, including walking, sitting, and standing up from a chair [1]. Typical hip–knee–ankle–foot (HKAF) prostheses require greater effort compared to lower-level amputations to perform these activities, especially for individuals who may lack the necessary physical fitness [2,3].

In individuals with transfemoral (TF), HD, or HP amputations, prosthetic joints provide minimal moment assist during sit-to-stand and stand-to-sit transitions [4]. Consequently, users rely on compensatory loading of the intact limb [5] and external aids, such as armrests [4]. For TF users, balance is compromised by excessive medial–lateral center of mass (COM) displacement and prolonged task duration. Reliance on the intact limb for propulsion and descent shifts the COM laterally [6], exiting the base of support early and increasing vulnerability to disturbance [7]. Furthermore, limited prosthetic moment generation and the damping resistance of microprocessor-controlled knees slow transition times compared to able-bodied controls. This prolonged duration increases the risk of failed push-offs and falls [5,8].

Recent advancements in motorized prosthetic knee joints demonstrate the potential benefits of active components in prosthetic devices. Motorized knee joints provide better kinetic symmetry and enhanced control during demanding activities compared to microprocessor-controlled and passive knee joints [9,10,11]. During sit-to-stand and stand-to-sit, motorized knee joints generate net-positive torque, which increases internal joint moments and reduces the necessary compensation by the intact limb. Improved kinetic symmetry can alleviate physical strain and improve balance control during these tasks [12]. Furthermore, motorized prostheses may reduce the physical demands required to operate the device, thereby increasing independence for less active users [13].

Sit-to-stand and stand-to-sit are particularly demanding for HKAF prosthesis users [1]. However, compared to the advancements in prosthetic knees, there are currently no biomechanical studies characterizing these transitions in the HKAF population, nor have any methods been investigated to mitigate the physical effort required. The total absence of data prevents the development of evidence-based solutions. Given the success of active actuation in knees, a powered prosthetic hip joint utilizing similar technology presents a promising, yet unexplored, solution to reduce physical demand and prevent failed transition attempts [14].

In this feasibility study, we presented the development and initial evaluation of a control strategy for a motorized HKAF prosthesis during sit-to-stand and stand-to-sit transitions. The primary objective was to assess the feasibility of active hip actuation to determine its potential for prosthetic-side assistive torque generation, reducing intact-limb loading, and shorter task duration relative to conventional passive devices. The findings from this study will contribute to our understanding of the effectiveness of motorized hip joints in bilateral HKAF prosthesis users. Moreover, this research may stimulate further innovations to improve the quality of life for individuals with HD or HP amputations.

## 2. HKAF Prosthesis Prototype (Power Hip)

**Figure 1:**
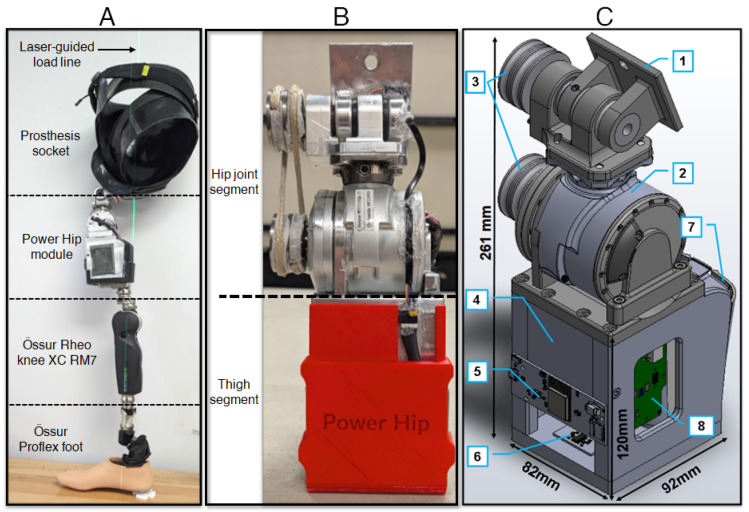
(**A**) Fully assembled Power Hip HKAF prototype, (**B**) Frontal view of Power Hip module, (**C**) Power Hip components, 1: interface plate between the Power Hip and prosthetic socket lamination plate. 2: DC motor, 3: motor torque transfer pulleys. 4: thigh chassis frame, 5: Sensor signal processing and active control electronics, 6: Load cells, 7: battery, 8: motor driver electronics.

The HKAF prosthetic prototype shown in Figure 1 was developed to enable walking, sitting, and standing for HD amputees. The prototype consists of an HD socket, a prototype front-mountable Power Hip, an Össur Rheo knee XC RM7 joint, and an Össur Pro-Flex foot. Building upon previous research [15,16,17], the Power Hip module design was further optimized for this study by reducing the overall length to accommodate people with shorter legs while ensuring compatibility with the existing HD socket designs and conventional prosthesis alignment methods [18].

The Power Hip module comprises two segments: the hip joint and the thigh. The hip joint features a single-axis joint that can transmit motor-induced torque to the anterior-mounted prosthetic hip centre of rotation. The thigh segment houses the electronics, sensors, and a battery. Due to space constraints near the mounting point, the actuator was positioned further below the socket, and torque transfer to the joint center of rotation was achieved using a cross-belt pulley system [19]. The actuator is a repurposed Össur Power Knee (Gen 2) three-phase brushless DC motor equipped with a harmonic drive gear system, capable of achieving a rotation velocity of 300°/s and supporting hip moment generation for users weighing up to 100 kg. The Power Hip was developed as a self-contained and untethered system. All sensing, data processing, and power delivery operations are performed in real-time by the integrated microcontroller and motor driver electronics inside the prosthesis, eliminating the need for external wiring to a computer or external power source. A summary of the Power Hip module mechanical characteristics is presented in Table 1.

## 3. Control Strategy

The hierarchical control strategy developed for Power Hip gait control in [20] did not include sit-to-stand and stand-to-sit transitions. The control strategy developed in this paper extends the control strategy discussed in [20] by integrating Bluetooth communication to enable real-time activation of activity sequences and by developing two new sets of finite state machines to decompose sitting/standing activities into a series of shorter phases.

The prototype sit–stand control uses custom graphical user interface software, running on a separate PC, to transmit activity execution requests to the prosthetic control system. Upon receiving these requests, the Power Hip module emits an audible beep, signaling to the user that it is ready to perform the requested activity. This signaling mechanism was implemented to minimize the risk of unintentional activation in the prototype system. Although the Bluetooth communication method was used for this purpose, other methods, such as double-tapping the prosthetic socket or using a remote-control dongle, could also be considered for future applications.

**Figure 2:**
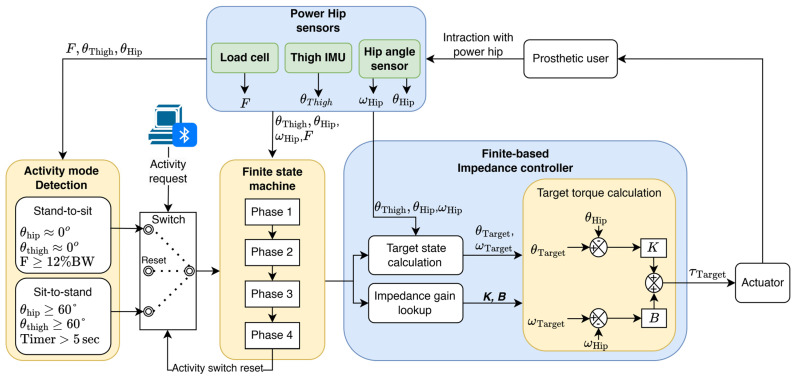
Overview of Power Hip control strategy for sit-to-stand and stand-to-sit.

As shown in Figure 2, when a sit or stand execution request is sent to the Power Hip, the control software first evaluates the user’s initial posture (idle sitting or standing) using hip angle sensors, a thigh inertial measurement unit (IMU) sensor, and the strain-gauged chassis (axial force sensor). If the user is in idle sitting mode, the sit-to-stand control protocol is executed to assist the user during standing. Conversely, if the user is in idle standing mode, the stand-to-sit protocol is activated to facilitate sitting. Idle sitting is determined when the hip flexion/extension angle ($\theta_{Hip}$) and the prosthetic thigh sagittal tilt angle ($\theta_{Thigh}$) remain greater than or equal to 60° for more than five seconds. Idle standing is determined when the hip is at an approximate equilibrium angle (${-1{}^{\circ}\leq \theta }_{Hip}\leq 1{}^{\circ}$, ${-1{}^{\circ}\leq \theta }_{Thigh}\leq 1{}^{\circ}$) and ground reaction force (GRF) exceeds 12% of the prosthetic user’s body weight when the request signal is received.

Regulation of Power Hip assistive and damping torques for both activities was performed by a finite-state-based impedance controller. This type of controller has been widely implemented in motorized transfemoral prostheses due to its simplicity and adaptability [21,22]. Finite-based impedance controllers operate by decomposing activities into smaller, sequential phases. Specific algorithms and gain values are applied for each phase to adjust the prosthesis angle and velocity impedance (magnitude of joint stiffness) [23].

In this study, the phase-based decomposition approach introduced by Kerr et al. [24] was adopted for the Power Hip impedance controller. Sit-to-stand and stand-to-sit activities were divided into four distinct chronological phases. Progression between phases occurs only when predefined transition conditions are met. At the start of each phase, the target hip angle ($\theta_{Target}$), target hip angular velocity ($\omega_{Target}$), velocity gain (B), and displacement gain (K) are computed and then placed into the motor torque equation:

(1) $$\tau_{Target}=\left(\theta_{Hip}-\theta_{Target}\right)K+\left(\omega_{Hip}-\omega_{Target}\right)B$$

where $\theta_{Hip}$ is the hip angle relative to the socket, $\omega_{Hip}$ is the measured hip angular velocity, and $\tau_{Target}$ is the calculated target torque for the Power Hip motor. Depending on the mathematical sign of $\tau_{Target}$ and $\omega_{Hip},$ Power Hip may dampen or assist movement. When $(\tau_{Target}/\omega_{Hip})\;<0$, Power Hip produces a damping torque to slow down or halt actuation. When $(\tau_{Target}/\omega_{Hip})\;>0$, Power Hip produces assistive torque to accelerate or maintain the velocity of hip joint actuation.

$\theta_{Target}$ and $\omega_{Target}$ are dynamically calculated for each phase based on the current and past states of $\omega_{Hip}$, $\theta_{Hip}$, and $\theta_{Thigh}$, while the B and K gains are manually tuned during participant training sessions to adjust for velocity and displacement impedance, respectively. The algorithms for computing the target states and the procedures for tuning B and K gains for each phase are detailed in the following subsections.

### 3.1. Sit-to-Stand Control Phases

**Figure 3:**
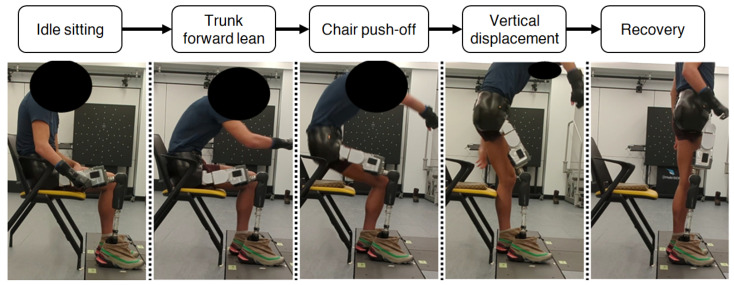
Decomposition of sit-to-stand for an HD participant using Power Hip HKAF prosthesis.

Observations of HD participant kinematics showed that their sit-to-stand transitions with mechanical hip joints were performed comparably to those of TF amputees [5,24]. Since active hip torque generation is required throughout the sit-to-stand motion, passive hip joints cannot provide any assistance to the user, resulting in minimal loading on the prosthetic side. As shown in Figure 3, sit-to-stand can be decomposed into four main phases: trunk forward lean, chair push-off, vertical displacement, and recovery [5].

#### 3.1.1. Phase 1: Trunk Forward Lean

Sit-to-stand initiation involves trunk forward leaning. This motion reduces the distance between the COM and the base of support, thereby decreasing hip and knee extension moments during the subsequent chair push-off (Phase 2) [25].

During Phase 1, the Power Hip provides minimal assistance to the user. Instead, a small damping torque is applied to enable the force sensors to detect COM movement as soon as the trunk begins to lean forward. This damping torque is achieved by setting the target hip angle ($\theta_{Target}$) to 90° and maintaining the displacement gain (K) at 0 < K ≤ 1. The velocity gain (B) is set to zero since controlling the velocity during this phase would not provide any benefit.

The transition from Phase 1 to Phase 2 is detected by the Power Hip when the COM is sufficiently close to the base of support to allow for assisted push-off [26]. Hence, the ground reaction force (F), measured by Power Hip, must exceed 2% of the body weight (BW), and the hip angle ($\theta_{Hip}$) must meet or exceed the hip extension threshold angle $\theta_{ST\_Phase1}$. The threshold $\theta_{ST\_Phase1}$ is an adjustable parameter that is tuned during prosthesis fitting and training.

#### 3.1.2. Phase 2: Chair Push-Off

Chair push-off transfers body weight as fast as possible from the chair to the feet by moving the COM toward the base of the support area. Immediately following the transition to Phase 2, a substantial hip extension moment is applied while both knees are loaded within a short period. This generates a downward force on the ground (action), facilitating COM ascent (reaction) [26]. During this phase, the Power Hip assists the user by producing a high assistive hip extension torque. This is achieved by setting stiffness K≥5 N·m/deg and B≥ 2 N·s/m at a controlled target angle $\theta_{Target}=0{}^{\circ}$ and a target angular speed ($\omega_{Target\;})$ defined as

(2) $$\omega_{Target}=-\left|\omega_{ST\_Phase1}\right|\times {∁}_{ST\_Phase2}$$

where $\omega_{ST\_Phase1}$ represents the peak hip flexion speed measured during Phase 1 and ${∁}_{ST\_Phase2}$ is a participant-specific tunable linear scaling factor. Since the Power Hip joint extends during Phase 2, the $\omega_{Target\;}$ must always be negative.

**Figure 4:**
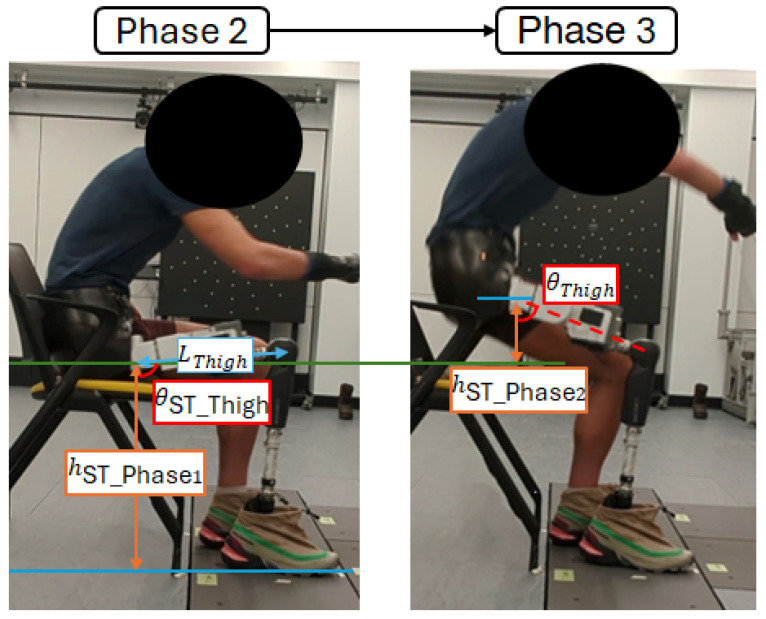
Visualization of chair push-off parameters.

The transition from Phase 2 to Phase 3 occurs when sufficient propulsion height is achieved. This height can be calculated using the thigh angle ($\theta_{Thigh}$) measured by the Power Hip IMU. Hence, transition completion can be determined when

(3) $$\left(L_{Thigh\;}\times \sin\left(\theta_{\mathrm{ST}\_\mathrm{Thigh}}-\theta_{Thigh}\right))\geq {(h}_{\mathrm{ST}\_\mathrm{Phase}2}+h_{\mathrm{ST}\_\mathrm{Phase}1}\right)$$

where as illustrated in Figure 4, $L_{Thigh}$ is the length of the Power Hip module, $\theta_{\mathrm{ST}\_\mathrm{Thigh}}$ is the thigh angle during idle sitting, $h_{\mathrm{ST}\_\mathrm{Phase}1}$ is the calculated hip joint height during Phase 1 of sit-to-stand, and $h_{\mathrm{ST}\_\mathrm{Phase}2}$ is the tunable target propulsion height.

#### 3.1.3. Phase 3: Vertical Displacement

During Phase 3, the trunk begins to lean anteriorly while the hip continues to extend. At this stage, the high assistive torque used during push-off propulsion is no longer required since the COM is already in motion and remains within the base of support after completing Phase 2. Instead, the Power Hip generates a small assistive torque to support continued hip extension and upward COM movement. This is achieved by setting torque parameters to 1 < K < 10 N·m/deg, B ≤ 1 N·s/m, and $\theta_{Target}=0{}^{\circ}$.

For a successful sit-to-stand completion, the hip joint must continue extending ($\omega_{Hip}\leq \;0$) since any hip flexion during this phase results in COM descent, potentially causing the user to fall back into a seated position [7]. To prevent this, the Power Hip control locks in position when a downward COM displacement is detected (when $\omega_{Hip}>0{}^{\circ}/s$). COM displacement direction-dependent torque control was implemented by alternating between high-damping torque and low assistive torque based on the $\omega_{Hip}\;direction$. High damping torque is applied by setting K > 100 N·m/deg, B = 0 N·s/m, and $\theta_{Target}=\theta_{ST\_Phase3}$, where $\theta_{ST\_Phase3}$ is the last measured hip angle while $\omega_{Hip}<0$.

Phase 3 is concluded when the prosthetic thigh’s absolute tilt angle reaches zero while $\theta_{Hip}>0$, indicating that the maximum COM height is achieved while prosthetic knee and hip joints are in flexion.

#### 3.1.4. Phase 4: Sit-to-Stand Recovery

At the start of Phase 4, both prosthetic hip and knee joints are in flexion, and therefore hip extension must be performed toward the equilibrium angle to ensure COM stability [7] (i.e., prosthetic knee positioned directly beneath the prosthetic socket at its maximum extension angle to lock the knee in stance mode and prevent accidental knee buckling [27]). To facilitate this motion, the Power Hip generates a high assistive hip extension torque, achieved by setting K>10 N·m/deg, B=0 N·s/m, and $\theta_{Target}=0{}^{\circ}$. This high assistive torque drives the $\theta_{Hip}$ toward the equilibrium position. Through the coupled kinematics of the Power Hip and prosthetic knee, this action simultaneously rotates the knee joint into full extension, ensuring stability and readiness for subsequent activities.

Once the $\theta_{Hip}$ reaches equilibrium, a high-damping torque is continuously applied to maintain position. This is necessary because the Power Hip extension range is larger than the range for mechanical hip joints. In mechanical hip joints, the joint is physically stopped against the socket in the standing position, whereas Power Hip enables a full extension range of motion, −10° to −25°, thereby requiring active damping control in equilibrium.

### 3.2. Stand-to-Sit Control Phases

The progressive stance-controlled resistance of some prosthetic knees can pose challenges for TF and HKAF prosthetic users during the stand-to-sit transition [9]. Users often rely on complementary maneuvers to generate sufficient hip and knee flexion moments throughout the activity [28]. The Power Hip joint provides an advantage in this context by producing assistive and damping torque, thereby minimizing the need for prosthetic side unloading or compensatory maneuvers.

**Figure 5:**
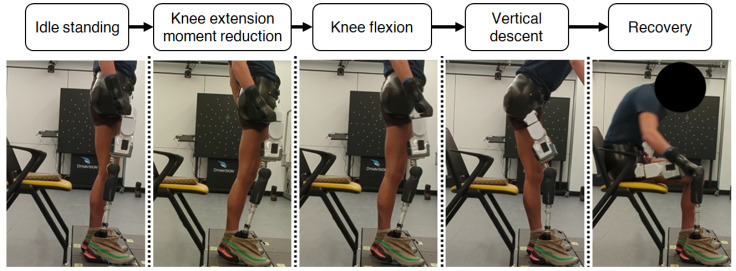
Stand-to-sit phase-based decomposition for Power Hip HKAF prosthesis.

Kerr et al. [24] previously decomposed the stand-to-sit activity of non-amputees into four distinct phases: trunk forward lean, knee flexion, vertical descent, and recovery. For the Power Hip, the decomposition was slightly modified to represent the observed kinematic characteristics (Figure 5). In preparation for knee flexion, instead of performing trunk forward leaning, HKAF users are required to move their prosthesis anteriorly to reduce the prosthetic knee extension moment. Therefore, trunk forward leaning during Phase 1 was replaced with “Knee extension moment reduction” to account for the differences in strategy.

#### 3.2.1. Phase 1: Knee Extension Moment Reduction

During idle standing, microprocessor-controlled knee joints generate high extension damping torque to prevent accidental buckling. However, this safety feature becomes a limitation during the stand-to-sit transition since this approach increases the minimum torque required to initiate knee flexion [9]. Participant testing revealed that the torque required at the hip to counter the knee extension moment exceeded the Power Hip motor’s maximum torque capability (96 N·m). This necessitated the development of a new strategy for prosthetic users to reduce the knee extension moment.

Since the magnitude of the knee extension moment depends on the load applied to the prosthetic side and the load’s horizontal distance from the knee’s center of rotation, two possible strategies to reduce this moment are to reduce prosthetic-side loading or shift the COM posteriorly. While unloading the prosthetic side can reduce the knee extension moment, this alters the base of support and decreases kinetic symmetry during the transition. Therefore, an alternative strategy was devised through participant testing to rely only on posterior COM movement.

During Phase 1, the Power Hip maintains a steady damping torque near equilibrium (K > 10 N·m/deg, B ≤ 1 N·s/m, and $\theta_{Target}=0{}^{\circ}$) while the participant uses their pelvis to perform rapid hip extension resembling a kicking motion. This maneuver momentarily (<1 s) shifts the COM posterior to the knee, increasing the moment arm length and thereby reducing the knee extension moment. The Phase 1 to Phase 2 transition is complete when rapid hip extension is detected. Hence, the transition condition is $\omega_{Hip}<\omega_{SI\_Phase1}<0{}^{\circ}/s$, where $\omega_{SI\_Phase1}$ is a tunable hip extension velocity threshold.

#### 3.2.2. Phase 2: Knee Flexion

By leveraging the reduced knee extension moment achieved during Phase 1 via chained kinematics, assistive hip flexion torque is sufficient to initiate knee flexion. Using the Power Hip, this is achieved by configuring Equation (1) parameters as K > 10 N·m/deg, B = 0 N·s/m, $\theta_{Target}=\theta_{SI\_Phase2}\geq 10{}^{\circ}$, where $\theta_{SI\_Phase2}$ is the tunable hip max flexion angle to be achieved during Phase 2.

To prevent balance loss during the kicking motion in Phase 1, hip flexion is performed immediately upon transitioning to Phase 2. This motion is delivered as a brief hip flexion torque burst to initiate the COM descent required for Phase 3. The transition from Phase 2 to Phase 3 is completed once $\theta_{Hip}\geq \;\theta_{SI\_Phase2}$.

#### 3.2.3. Phase 3: Vertical Descent

During vertical descent, the user initiates intact side hip and knee flexion, followed by a forward trunk lean, which prepares the user for body weight transfer onto the seat. At this phase, the Power Hip assists the user by providing assistive hip flexion to continually apply extension moments onto the knee, resulting in prosthetic knee flexion [12]. This assistive torque is calculated by setting K ≥ 1 N·m/deg, B ≥ 1 N·s/m, $\theta_{Target}=90{}^{\circ}$, and $\omega_{Target}$ = $\omega_{SI\_Phase3}$, where $\omega_{SI\_Phase3}$ is the hip flexion speed during stand-to-sit Phase 3 and is calculated using Equation (4),

(4) $$\omega_{SI\_Phase3}=\omega_{SI\_Phase1}\times {∁}_{SI\_Phase3}$$

where ${∁}_{SI\_Phase3}$ is a tunable ratio.

The transition from Phase 3 to Phase 4 is completed when the COM is a few centimetres above the seat. The distance can be estimated by measuring the thigh’s absolute tilting angle and assuming that the seat is at knee height. Hence, the transition condition is met when

(5) $$\theta_{Thigh}\leq \sin^{-1}(\frac{h_{\mathrm{SI}\_\mathrm{Phase}3}}{L_{Thigh}})$$

where $h_{\mathrm{SI}\_\mathrm{Phase}3}$ is an adjustable threshold for the minimum height of the prosthetic user socket relative to the seat.

#### 3.2.4. Phase 4: Stand-to-Sit Recovery

During this phase, the prosthetic user transitions into idle sitting by allowing their body to settle onto the seat. Power Hip does not assist during this phase, since neither displacement nor velocity control provides functional benefits. Consequently, no torque is produced by the Power Hip when the Equation (1) parameters are set to K = 0 N·m/deg and B = 0 N·s/m.

## 4. Power Hip Performance Evaluation

To assess the feasibility of the Power Hip, a single participant with HD was enrolled in an experimental protocol. The evaluation consisted of a training phase for sit-to-stand and stand-to-sit transitions, followed by the acquisition of kinematic and kinetic data through motion capture. The performance of the motorized system was benchmarked against the participant’s prescribed passive HKAF prosthesis, with a focus on task execution duration and bilateral kinetic symmetry as key indicators of feasibility.

### 4.1. Participant Recruitment and Preparation

This study was approved by the University of Ottawa (H-08-21-7062) and Carleton University (122696) ethics boards. Inclusion criteria required participants to have unilateral hip disarticulation (right side), a minimum of three months of experience using a HKAF prosthesis, be at least 18 years old, have a body weight under 100 kg (without the prosthesis), lead an active lifestyle, use their prosthesis daily, and have the ability to ambulate independently without assistive devices such as a cane. A male participant who met these criteria was recruited for the study (height = 1.80 m, age = 25 years, mass = 64 kg, prosthesis experience = 6 years). His current prosthesis had an Ottobock Helix3D hip joint and Ottobock Genium knee. He was fitted with a new hip disarticulation socket replicated by a certified prosthetist from his prescribed socket at Nielen Prosthetic and Orthotic Clinic (860 Campbell Ave, Ottawa, ON, Canada, K2A 2C5).

Participant trained performing sit-to-stand and stand-to-sit activities with Power Hip in ten sessions, each lasting one hour. During the training period, the control strategies were refined, and tunable control parameters were adjusted based on the participant’s feedback and prosthetist observations. The training was deemed complete once the participant successfully executed five consecutive sit–stand–sit transitions.

### 4.2. Test Protocol

Following completion of training, evaluation trials were conducted at the Abilities Living Laboratory (Carleton University, Ottawa, Canada). Whole-body three-dimensional movements were recorded using 11 high-definition cameras operating at 100 Hz. The recorded videos were processed into a 13-segment, six-degrees-of-freedom skeletal model pose using the Theia3D markerless motion capture system (version 2024.1.0.4409). The pose data were smoothed using a generalized cross-validation spline method with a 20 Hz cutoff frequency [29]. Ground reaction forces were collected with two force plates (AMTI BMS400600) at a sampling rate of 1000 Hz and synchronized with the cameras via OptiTrack eSync2 hardware and OptiTrack Motive software (version 3.1.0). Force plate signals were subsequently filtered using a fourth-order Butterworth low-pass filter with a 10 Hz cutoff frequency.

Prior to testing, the system was calibrated according to Theia3D documentation [30]. After calibration, a chair with armrests was positioned in front of the force platforms. Then, the participant was asked to sit on the chair with one foot on each force plate. Ten sit–stand–sit trials were completed in one session: five with their prescribed prosthesis and five with the Power Hip. With Power Hip, sit-to-stand and stand-to-sit modes were activated sequentially via Bluetooth commands sent from a custom-built personal computer application. A minimum of 10 s was provided between each sit–stand–sit to allow the participant to adjust their posture and reestablish their idle posture (standing or sitting).

### 4.3. Data Analysis

The 3D model generated by Theia3D was imported into Visual3D (version 2024.07.2) to compute lower extremity joint kinetics and kinematics. Hip and knee joint angles were calculated using a Cardan sequence [31]. Trunk COM movements were calculated from the body model (in reference to the lab coordinate system) to identify movement onset and cessation for the derivation of temporal outcome measures. Joint moments and resultant GRF were calculated through inverse dynamics.

Each trial’s data were segmented according to the activity type (sit-to-stand or stand-to-sit) using the method described by Highsmith et al. [12]:

- **Sit-to-stand**: initiation occurs when the trunk COM moves forward by 10 mm and is completed when both the hip and knee are fully extended and remain extended for more than 0.25 s (±2° margin of error).
- **Stand-to-sit**: initiation occurs when either the hip or knee (prosthetic side) flexes by more than 5° relative to the idle standing equilibrium angle and is completed when the trunk COM ceases posterior movement for more than 0.25 s (±5 mm margin of error) and both the hip and knee are flexed beyond 60°.

The segmented kinetic and kinematic data were exported to MATLAB (R2024a) for further analysis and calculation of outcome measures. Primary outcome measures included maximum resultant GRF, resultant GRF impulse, maximum knee moment, maximum hip moment, sit-to-stand duration, and stand-to-sit duration. GRF was normalized by body weight. Knee and hip moments (N·m) were divided by the product of participant body mass and height (kg·m). Additionally, to quantify the intact and prosthetic side kinetic distribution across outcome measures, the following ratio equation was used:

(6) $$kinetic\;ratio=\left|\frac{prosthetic\;side\;kinetic\;outcome}{intact\;side\;kinetic\;outcome}\right|$$

where a higher ratio would indicate a more symmetrical distribution between the intact and prosthetic sides.

Each outcome measure consisted of a total of 20 samples. These were first categorized by the prosthesis type (Power Hip and prescribed HKAF), with 10 samples attributed to each. The data were further divided by activity mode (sit-to-stand and stand-to-sit), resulting in four distinct groups, each containing 5 samples. To compare the performance between activity modes and prosthetic types, the mean and standard deviation were computed for each of the four groups of each outcome measure.

The control strategy was designed to ensure that the prosthetic user maintained consistent prosthetic side loading while sitting and standing. Additionally, assistive hip torque was intended to counteract the prosthetic knee’s progressive resistance, thereby facilitating faster movements. Considering these capabilities, the Power Hip performance metrics should indicate shorter task execution times, reduced maximum kinetic differences between the intact and prosthetic sides, and higher kinetic ratios compared with prescribed prosthesis results, as observed in TF amputees using microprocessor-controlled knees versus motorized knees [5,12].

## 5. Results

The participant successfully completed all trials with both prostheses without losing balance or requiring external assistance. He used the chair armrests during push-off phases for both prostheses. The tunable parameters were initially configured to default values that were deemed non-taxing for prosthesis users. As the participant gained proficiency with the Power Hip during training, both finite state machine parameters and impedance controller gains were iteratively adjusted to optimize performance. The finalized parameter configurations are documented in Appendix A.

**Figure 6:**
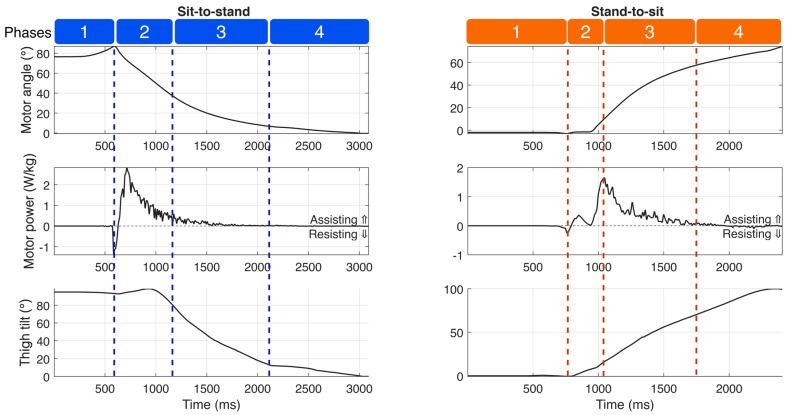
Power Hip sensor measured motor angle, motor power, and sagittal thigh segment tilt angle during one sitting and standing trial.

**Figure 7:**
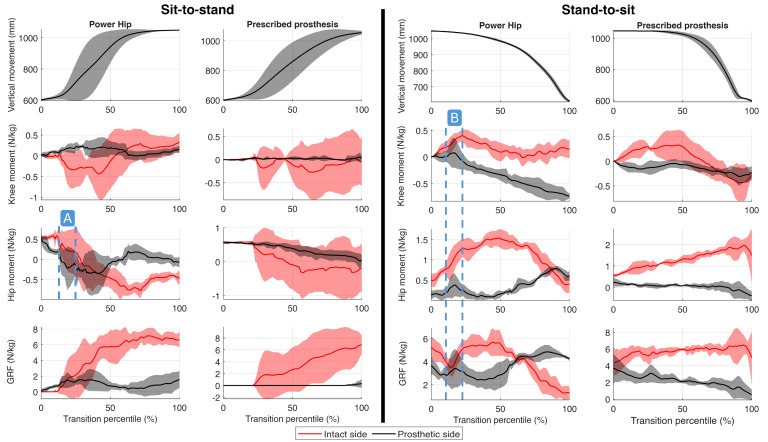
Kinetics for sit-to-stand and stand-to-sit with Power Hip and participant’s prescribed passive prosthesis (average and standard deviation). Positive values for the knee and hip joints indicate external moments in the flexion direction; negative values indicate external moments in the extension direction. Vertical movement represents the Z-axis movement of the pelvis COM. (**A marker**) The period representing Power Hip extension to assist during chair push-off (sit-to-stand Phase 2), (**B marker**) the period representing knee extension moment reduction (stand-to-sit Phase 1).

Sensor measurements from the Power Hip prosthesis are presented in Figure 6, while maximum kinetic values (hip joint moments, knee joint moments, GRF) are summarized in Table 2. Kinetic profiles and COM trajectories are illustrated in Figure 7. The positive knee and hip moments indicated external moments applied in the flexion direction, and negative moments indicated external extension moment generation.

### 5.1. Power Hip Motor Output Measurement

The Power Hip prosthesis executed all sit-to-stand and stand-to-sit transition phases. As shown in Figure 6, during sit-to-stand transitions, the prosthesis generated short, high-magnitude bursts of assistive power, peaking at 3.73 ± 0.35 W/kg during Phase 2 (chair push-off). In contrast, stand-to-sit transitions produced lower peak assistive power (1.77 ± 0.15 W/kg), which occurred during Phase 3 (vertical descent).

Figure 7A illustrates the effect of the Power Hip’s extension moment during Phase 2 of the sit-to-stand transition (chair push-off), corresponding to the assistive power that assisted in initiating the COM upward movement. Figure 7B shows the hip and knee flexion external moments during Phase 2 of the stand-to-sit transition, reflecting the prosthesis’s ability to reduce knee extension moments through active hip flexion.

### 5.2. Ground Reaction Force

During both sit-to-stand and stand-to-sit transitions, the participant exhibited a preference for loading the intact limb, consistent with patterns observed in TF amputees [12]. However, the Power Hip prosthesis reduced the maximum force disparity between the prosthetic and intact sides during transitions, with differences of 5.34 ± 0.68 N/kg during sit-to-stand and 1.00 ± 0.64 N/kg during stand-to-sit. These improvements were attributed to peak kinetic outputs facilitated by the Power Hip’s actuation bursts during early transition phases (Figure 7A,B). In contrast, the prescribed prosthesis resulted in larger force differences (7.34 ± 1.87 N/kg during sit-to-stand; 3.70 ± 1.95 N/kg during stand-to-sit).

Similar differences were also observed with GRF impulses. With the prescribed prosthesis, sit-to-stand transitions showed intact-side dominance, with impulses of 4.16 ± 4.02 N·s/kg (intact) versus 0.03 ± 0.07 N·s/kg (prosthetic). The Power Hip promoted more balanced loading, yielding 7.91 ± 3.58 N·s/kg (intact) and 1.66 ± 1.10 N·s/kg (prosthetic). Similarly, during stand-to-sit transitions, the Power Hip reduced GRF impulse differences (0.61 ± 0.83 N·s/kg) compared to the prescribed prosthesis (9.37 ± 2.69 N·s/kg).

Maximum summed GRF magnitudes during sit-to-stand transitions were lower than the participant’s body mass (64 kg) for both prostheses (Power Hip: 55.94 ± 3.62 kg; prescribed: 49.88 ± 13.30 kg).

### 5.3. Hip Moment

Hip joint moment differences between intact and prosthetic sides were greatly reduced with the Power Hip prosthesis compared to the prescribed HKAF prosthesis during both sit-to-stand (Power Hip: −0.18 ± 0.09 N/kg, prescribed: −0.69 ± 0.67 N/kg) and stand-to-sit (Power Hip: 0.77 ± 0.20 N/kg, prescribed: 2.03 ± 0.58 N/kg).

During sit-to-stand, the Power Hip generated a maximum hip extension moment of −0.79 ± 0.06 N/kg, whereas the prescribed joint dampened hip movement by generating a small flexion torque (0.01 ± 0.21 N/kg). However, during stand-to-sit transitions, the prescribed HKAF appeared to contribute flexion moments, which were achieved through externally generated forces (e.g., using his hand to reposition the prosthetic limb and flex the knee manually) rather than intrinsic joint actuation. This differs from the Power Hip, which generated flexion moments via motor actuations.

### 5.4. Knee Moment

During sit-to-stand transitions, the Power Hip prosthesis generated a prosthetic-side knee flexion moment of 0.35 ± 0.02 N/kg, actively damping joint movement, which was higher than the prescribed HKAF prosthesis (0.09 ± 0.06 N/kg). Similarly, during stand-to-sit transitions, the Power Hip’s prosthetic knee produced a flexion moment of −0.48 ± 0.06 N/kg to dampen knee extension, compared to the minimal damping observed with the prescribed prosthesis (−0.10 ± 0.05 N/kg).

### 5.5. Duration

The participant completed both sit-to-stand and stand-to-sit transitions more rapidly with both prostheses, compared to average durations reported in prior literature on TF amputees (Highsmith et al. [12]). However, distinct temporal patterns emerged between HKAF prosthesis types. During sit-to-stand transitions, the Power Hip prolonged the transition (1.69 ± 0.49 s) relative to the prescribed HKAF (1.22 ± 0.40 s), though both remained faster than reported TF amputees (2.0 ± 0.8 s). In contrast, during stand-to-sit transitions, the Power Hip enabled faster transitions (1.22 ± 0.08 s) compared to both the prescribed HKAF (2.62 ± 0.41 s) and TF group (2.8 ± 0.6 s).

## 6. Discussion

This study demonstrated that a novel Power Hip prosthesis can enable more symmetric lower limb loading during sit-to-stand and stand-to-sit by providing appropriate hip moments at the right time, as evidenced by higher prosthetic/intact side ratios of resultant max GRF, max hip moment, and max knee moment. Furthermore, the stand-to-sit transition time was reduced. However, the sit-to-stand transition time was longer than that of conventional joints. This hip joint advancement has the potential to improve functional outcomes during sit-to-stand and stand-to-sit transitions in individuals with HD amputations.

### 6.1. Kinetic Differences

Analysis of kinematic data (Figure 7) and peak kinetic values (Table 2) demonstrated two principal advancements of the Power Hip over the prescribed prosthesis: improved weight-bearing on the prosthetic limb during transitions and reduced reliance on upper-body compensation.

The Power Hip suggested improvements in redistributing kinetic demands between limbs during sit-to-stand and stand-to-sit. The Power Hip reduced asymmetry in measured GRF and hip joint moments compared to the prescribed HKAF prosthesis. The Power Hip’s assistive hip extension moments during sit-to-stand and controlled flexion assistance during stand-to-sit replicated biomechanical patterns observed in TF amputees [32], thereby reducing intact side overloading.

The kinetic profiles in this study diverged from those reported in the TF amputees and able-bodied literature [33,34,35]. HD amputees inherently adopt distinct COM transitional strategies due to the absence of a femoral segment, which alters weight-shifting mechanics. Many existing biomechanical studies restrict participants from using external supports, such as armrests, during transitions. The allowance of armrest support in our study, therefore, likely contributed to the difference in kinetic profiles by enabling the participant to offload body weight and alter the required lower-limb moments. However, when comparing within the context of the HD participant, our preliminary kinematic and temporal data suggest that the Power Hip may reduce the need for compensatory reliance on upper-body support compared to the passive prosthesis.

During stand-to-sit with the prescribed HKAF, the participant was observed applying manual pressure to the prosthetic thigh to initiate hip and knee flexion during the chair push-off, counteracting the prosthetic knee’s extension moment. The Power Hip active flexion control eliminated the need for this manual intervention during the push-off phase. However, as the descent (Phase 3 of stand-to-sit) began, the participant consistently reached for the chair armrests for support regardless of the prosthesis type.

The temporal GRF data further supported the redistribution of kinetic demands. The Power Hip kinetics showed higher intact- and prosthetic-side GRF values from the onset of Phase 1 of the sit-to-stand transition, suggesting that the lower limbs were engaged earlier in the transition. However, during prescribed HKAF trials, a period (20–40% of the transition) occurred during which negligible GRF was recorded on either limb, even as the COM was displacing vertically (Figure 7). This absence of reaction force implies near-total dependence on the upper body and armrests to support body weight during that specific interval. Future studies utilizing instrumented armrests or upper-body EMG are necessary to quantify the exact magnitude of this compensatory effort.

The high prosthetic knee damping during Power Hip stand-to-sit stemmed from the Rheo Knee 3 progressive stance control feature that resisted knee movement. The limited moment generation of the prescribed prosthetic knee could have been due to the limited prosthetic-side loading since prosthetic knees are designed to dampen only when they are loaded beyond a certain threshold [32].

### 6.2. Transition Execution Time

The Power Hip demonstrated contrasting performance profiles during sit-to-stand and stand-to-sit compared to the prescribed HKAF and TF amputee results from the literature [12]. During sit-to-stand, the participant achieved faster transitions with the prescribed HKAF (1.22 ± 0.40 s) than with Power Hip (1.69 ± 0.49 s). However, the prescribed HKAF reduced timing came at the cost of near-complete reliance on upper-body force generation, since the participant failed to load the prosthetic limb during chair push-off (Figure 7). In contrast, Power Hip promoted bilateral limb loading, reducing upper-body dependence and offering potential advantages for users with limited upper-limb strength.

Although increasing the Power Hip’s extension control gains could theoretically accelerate chair push-off, the participant reported pelvic discomfort due to abrupt torque transmission when gains were excessive. In future iterations of the sit-to-stand control strategy, this limitation could be addressed by smoothing torque profiles or by using control algorithms that more accurately estimate the user’s desired push-off acceleration.

The participant’s faster overall transition times relative to the TF amputees may stem from methodological differences, particularly the permitted use of chair armrests in this study. Such external assistance likely reduced biomechanical demands, masking the full effect of prosthetic design on transition timing.

### 6.3. Limitations

The findings presented here should be considered within the constraints of the study design. The primary limitation stems from the single-user design; the participant was an experienced prosthesis user with an active lifestyle. Therefore, the results are preliminary and may not be readily generalizable to the broader HD population, particularly those with lower activity levels or less experience. Furthermore, the data reflects the performance of the Power Hip after a brief training period. As with any novel prosthesis, subsequent experience in daily activities could lead to a better utilization of its advanced capabilities [36].

Additionally, the heterogeneity of prosthetic components restricts the generalizability of kinetic data. The study employed a specific component configuration, coupling the Power Hip with the Össur Rheo Knee XC RM7. Since this specific knee lacked a dedicated stand-to-sit damping profile, the active hip flexion provided by the Power Hip was necessary to regulate vertical descent. This functionality may differ when paired with other microprocessor-controlled knees (e.g., Össur Navii, Ottobock Genium X4) that feature integrated descent controls. Finally, all testing was performed under controlled laboratory conditions, which included a fixed chair height, and armrest usage was allowed but not instrumented. These environmental factors simplify the task compared to varied real-world settings. Future research should assess performance across diverse chair heights and incorporate a larger, more diverse cohort using instrumented armrests to fully quantify compensatory effort across different component designs.

## 7. Conclusions

Active actuation in the HKAF prosthesis improved kinetic symmetry and reduced stand-to-sit task duration compared to the passive condition. The device successfully mimicked physiological hip kinematics by generating active moments. The participant’s ability to control the device with minimal training further supports the potential of powered hip joints to enhance transition performance for HD amputees.

However, challenges remain. The influence of external factors, such as chair height and upper-body assistance, on prosthetic performance warrants further investigation to improve control strategies. Future studies should explore how the Power Hip prosthesis can facilitate transitions without upper-limb support from armrests (a common requirement in real-world environments) and evaluate its efficacy across varied seating configurations. Such research would clarify the Power Hip’s adaptability to diverse user needs and environments. Ultimately, this research advances the design and evaluation of motorized prosthetic systems for HD amputees, offering a pathway to improved functional independence and quality of life.

## Figures and Tables

**Table 1:** Power Hip module mechanical characteristics.

Property	Value
Module dimensions	261 × 82 × 92 mm^3^
Module mass	3.1 kg
Joint pulley diameter	52 mm
Motor stall torque	96 N·m
Motor max speed	300°/s
Battery capacity	95.04 Wh
Motor router to shaft gear ratio	1:100

**Table 2:** Joint moments, resultant max ground reaction force (GRF) of each limb, GRF impulse, and execution duration for Power Hip and prescribed passive HKAF prosthesis during sit-to-stand and stand-to-sit transition. Ratios are the prosthetic side/intact side.

	Sit-to-Stand		Stand-to-Sit	
	Power Hip	Prescribed	Power Hip	Prescribed
Intact side resultant max GRF (N/kg)	8.03 ± 0.56	7.64 ± 2.04	6.37 ± 1.01	7.98 ± 1.03
Prosthetic side resultant max GRF (N/kg)	2.69 ± 0.34	0.30 ± 0.67	5.37 ± 0.52	4.28 ± 1.00
Resultant max GRF ratio	0.34 ± 0.05	0.03 ± 0.07	0.85 ± 0.09	0.56 ± 0.22
Intact side max GRF impulse (N·s/kg)	7.91 ± 3.58	4.16 ± 4.02	4.97 ± 0.78	15.06 ± 2.90
Prosthetic side max GRF impulse (N·s/kg)	1.66 ± 1.10	0.03 ± 0.07	4.36 ± 0.17	5.69 ± 0.66
Max GRF impulse ratio	0.20 ± 0.06	0.00 ± 0.01	0.90 ± 0.16	0.39 ± 0.08
Intact side max knee moment (N/kg)	−0.90 ± 0.19	−0.84 ± 0.32	0.69 ± 0.06	0.60 ± 0.26
Prosthetic side max knee moment (N/kg)	0.35 ± 0.02	0.09 ± 0.06	−0.48 ± 0.06	−0.10 ± 0.05
Max knee moment ratio	0.41 ± 0.08	0.12 ± 0.11	0.69 ± 0.04	0.19 ± 0.11
Intact side max hip moment (N/kg)	−0.97 ± 0.06	−0.68 ± 0.62	1.61 ± 0.18	2.35 ± 0.48
Prosthetic side max hip moment (N/kg)	−0.79 ± 0.06	0.01 ± 0.21	0.84 ± 0.06	0.32 ± 0.13
Max hip moment ratio	0.82 ± 0.08	0.03 ± 0.44	0.53 ± 0.08	0.15 ± 0.10
Transition duration (s)	1.69 ± 0.49	1.22 ± 0.40	1.22 ± 0.08	2.62 ± 0.41

## Data Availability

The datasets presented in this article are not readily available because the data are part of an ongoing study. Requests to access the datasets should be directed to fgolshan@uottawa.ca.

## Abbreviations

COM	Centre of mass
GRF	Ground reaction force
HD	Hip disarticulation
HKAF	Hip–knee–ankle–foot
HP	Hemipelvectomy
IMU	Inertial measurement unit
TF	Transfemoral

## Appendix A

List of tuned Power Hip sit-to-stand and stand-to-sit control parameters

Tunable control parameters:

- $\theta_{ST\_Phase1}=10$
- $h_{ST\_Phase2}=10$
- ${∁}_{ST\_Phase2}=0.6$
- $\omega_{SI\_Phase1}=-10$
- $\theta_{SI\_Phase2}=30$
- ${∁}_{SI\_Phase3}=3$
- $h_{SI\_Phase3}=10$

**Table A1:** Impedance based controller tunned gains for each sit-to-stand and stand-to-sit phases.

	Phase 1	Phase 2	Phase 3	Phase 4
**Sit-to-stand**				
K gain (N·m/deg)	0.2	5.0	10.0	23.0
B gain (N·s/m)	0.0	3.0	0.5	0.0
**Stand-to-sit**				
K gain (N·m/deg)	20.0	40.0	2.0	0.0
B gain (N·s/m)	1.0	0	1.0	0.0
